# Extrapulmonary COVID-19 Presents As Spontaneous Small Bowel Perforation

**DOI:** 10.7759/cureus.35524

**Published:** 2023-02-27

**Authors:** Stephanie N Brooks, Taylor Brown, Christopher Yeary

**Affiliations:** 1 Surgery, Kentucky College of Osteopathic Medicine, Pikeville, USA; 2 Internal Medicine, Norton Community Hospital, Norton, USA; 3 General Surgery, Norton Community Hospital, Norton, USA

**Keywords:** sars-cov2, enterectomy, small bowel perforation, pneumoperitoneum, covid-19

## Abstract

SARS-CoV2 is a well-recognized pathogen with a myriad of presenting symptoms. Well-documented pulmonary, neurological, gastrointestinal, and hematologic complications have occurred during the global COVID-19 pandemic. While gastrointestinal symptoms are the most commonly reported extrapulmonary symptom of COVID-19, the incidence of primary perforation has not been widely reported. In this case report, we describe a spontaneous small bowel perforation in a patient who was incidentally found to be COVID-19 positive. This peculiar case underlies the continued evolution of SARS-CoV2 understanding and potential unknown complications of the virus.

## Introduction

The typical COVID-19 presentation involving respiratory complaints has been well-defined since the start of the global pandemic in 2019 [1]. Extrapulmonary involvement and symptom presentation continue to be an area of active investigation as additional viral variants are discovered. The most commonly reported gastrointestinal symptoms in COVID-19 patients are nausea, vomiting, diarrhea, and abdominal pain [2]. Gastrointestinal perforation is a rare but life-threatening complication seen in COVID-19 patients due to ischemia, thrombosis, or the use of antiviral drugs [3]. We present a case of small bowel perforation, which was found to be positive for COVID-19 incidentally, with the only complaint of abdominal pain.

## Case presentation

**Figure 1:**
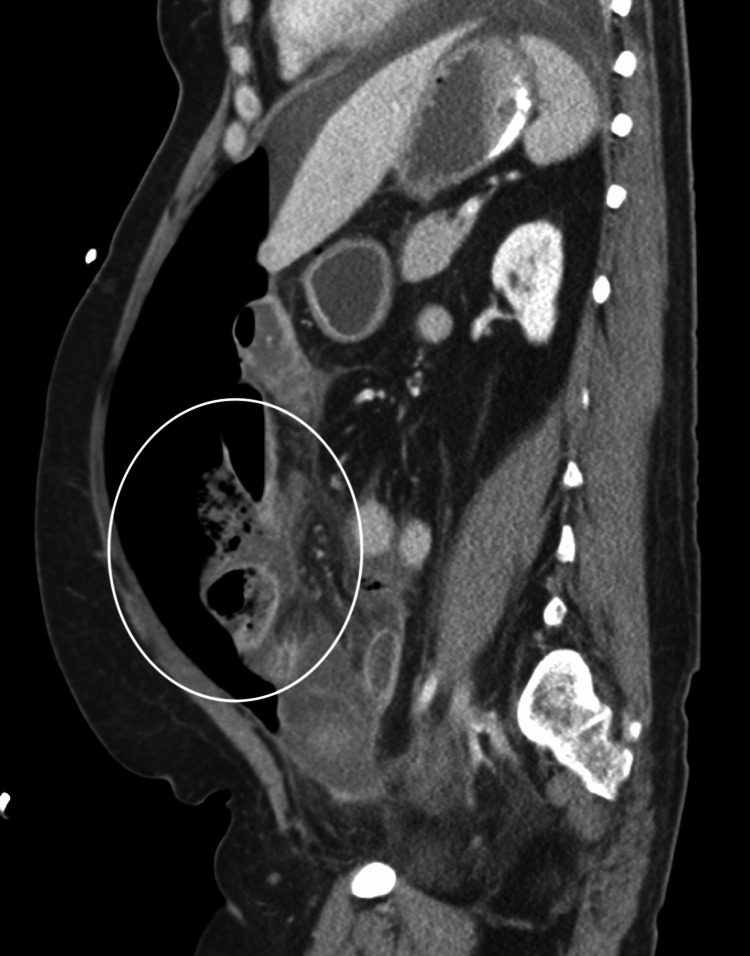
CT of the abdomen (sagittal view) showing pneumoperitoneum with significant abdominal distention.

**Figure 2:**
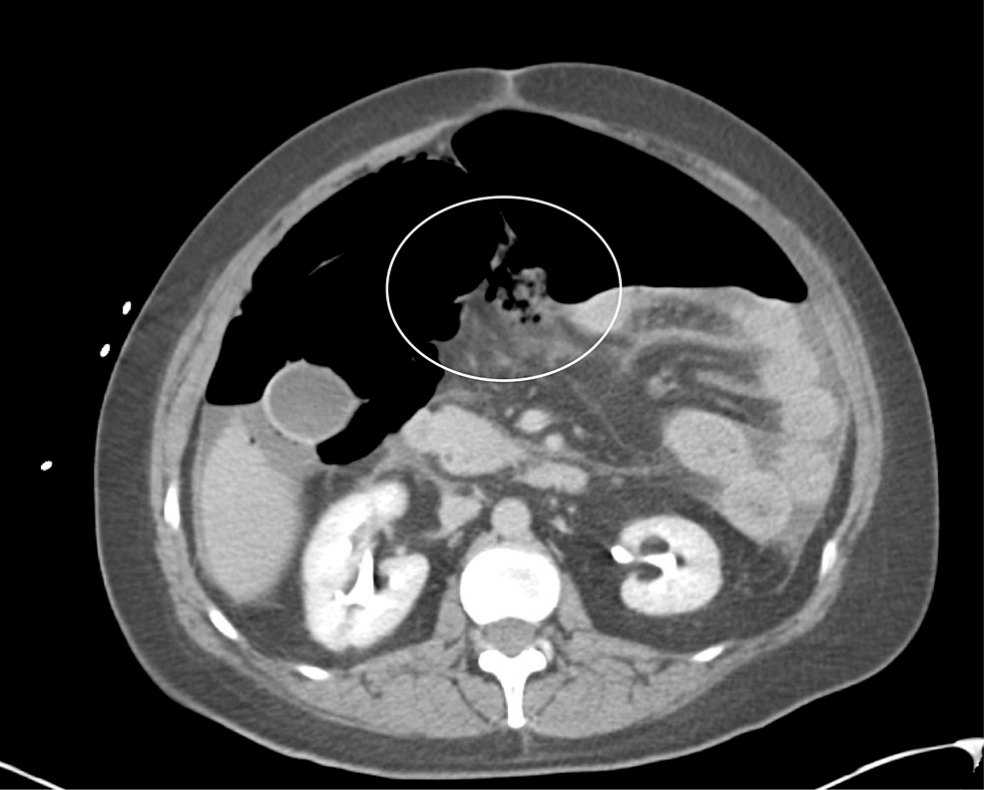
CT abdomen (axial view) showing distention and pneumotosis outside of preparation of small bowel.

A 46-year-old female presented to the emergency department via ambulance with acute abdominal pain. She reported diffuse bloating, burning, and sharp pain that did not radiate. The patient reported nausea and one episode of non-bloody, non-bilious emesis. She denied fever, chills, and cough. The patient has extensive previous abdominal surgical history, including subtotal colectomy with subsequent reversal 10 months prior due to ischemia. She also has a history of hypertension, depression, and anxiety. The patient was tested for COVID-19 using a rapid RT-PCR nasal swab, as this was the hospital policy for surgical patients. Incidentally, this was positive for SARS-CoV2. The patient had not yet received any COVID-19 vaccinations. The patient denied any respiratory symptoms, though she did report a history of cigarette use. However, the patient had no history of COPD or supplemental oxygen requirements. A computerized tomography (CT) of the abdomen and pelvis showed a large amount of free intraperitoneal air with a smaller amount of free fluid noted with extensive pneumatosis in the right abdomen involving the ileum (Figure 1, 2). The appendix was registered as poorly visualized, and diverticulitis was not identified on CT. Abnormal laboratory data was significant for leukocytosis (14,300 WBC/mm^3^) and elevated lactate (5.3 mm/L). Apparent peritonitis was present on physical examination.

General surgery was consulted, and the patient was taken to the operating room for exploratory laparotomy. Upon initial celiotomy, a solitary small bowel perforation with purulent peritonitis was appreciated, complicated by a mesenteric abscess adjacent to the perforation. The patient underwent an enterectomy without anastomosis. Given the patient's intestinal friability, peritoneal contamination, and hemodynamic instability, she was left in discontinuity with an application of Abthera Advance(TM) abdominal dressing. After appropriate resuscitation and global stabilization in the patient's medical condition, she was taken back to the operating room on a postoperative day 3 for staged exploration and closure. Her abdomen was washed out during the second look laparotomy, and no other pathology was appreciated. Anastomosis was avoided due to continued inflammation and friability of the rectal stump and a known history of recent cigarette use. Therefore, an end ileostomy was created. The patient was successfully extubated, with a return of bowel function and resumption of oral diet. She was discharged on postoperative day 11. The patient's course was complicated by re-admission for bacterial pneumonia on postoperative day 22, which was treated with IV antibiotics for 2 days in the hospital and discharged with a 6-day course of amoxicillin-clavulanate. 

The final pathology of the resected surgical specimen demonstrated a 2.5 x 1.3 x 0.8cm perforation of the small bowel. Pathology reported no ischemia, adenomatous changes, or malignancy of the specimen.

## Discussion

Causes of typical bowel perforations can be categorized into the following etiologies: ischemia, erosive, trauma, or infectious. Ischemic small bowel changes can be attributed to multiple overdistension modalities, causing mural ischemia to vascular insults. Erosive disease processes can occur from extrinsic pathologies such as an adjacent tumor or primary tumor invasion. Intrinsic disease, such as inflammatory bowel disease, can also be a factor. Traumatic disruption can occur due to mechanical injury to the bowel via blunt and penetrating abdominal injuries or iatrogenically. Infections resulting in small bowel perforations most commonly occur as a secondary sequela in the setting of other ongoing intra-abdominal infections, such as appendicitis or diverticulitis [4]. 

The most researched method of entry is the well-defined angiotensin-converting enzyme-2 (ACE-2) receptor, found in both pulmonary alveoli as well as the epithelium of the GI tract [5]. Multiple mechanisms of injury have been proposed for COVID-19's pathogenesis within the gastrointestinal tract, from ingestion of droplets to post-nasal drip. Fecal-oral transmission of the virus has also been offered due to the detection of viral RNA in stool samples [6]. The viral pathophysiology resulting in gastrointestinal perforations remains to be elucidated, which is undoubtedly made more challenging due to the rarity of these cases. 

Hollow viscus perforation events have been identified in COVID-19 patients. However, most cases currently described in the literature involve critically ill patients, specifically those with respiratory complaints and previously treated with steroids, Janus kinase 2 (JAK2) inhibitors, or Interleukin 6 (IL-6) inhibitors [3]. The use of either JAK2 inhibitors or IL-6 inhibitors is cautioned in patients with an increased risk for bowel perforation, such as those with a history of diverticulitis due to adverse events reported in clinical trials for chronic indications [7]. Our case is unique from those currently described in the literature, as the patient did not have respiratory symptoms, nor did she receive any treatment for COVID-19, including steroids or intravenous biologic therapy. The paucity of identifiable pathology on the final surgical specimen and lack of abdominal trauma leads us to suspect a diagnosis by excluding that infection; specifically, COVID-19 was the mechanism for this patient's bowel perforation. The patient's only complaint was acute abdominal pain, which was incidentally found to be COVID-19 positive due to hospital admission protocol.

## Conclusions

Hollow viscus abdominal perforations are a well known surgical emergency caused by multiple pathologies. In the absence of obvious etiologies, alternatives must be evaluated in cases of bowel perforation of unknown origin. Given the wide spectrum of presenting symptoms, COVID-19 should be considered as an infectious cause of spontaneous bowel perforation in a COVID-19 positive patient with an otherwise negative workup.
